# Recent progress in bio-mediated synthesis and applications of engineered nanomaterials for sustainable agriculture

**DOI:** 10.3389/fpls.2022.999505

**Published:** 2022-10-03

**Authors:** Kainat Amin Bora, Saud Hashmi, Faisal Zulfiqar, Zainul Abideen, Haibat Ali, Zamin Shaheed Siddiqui, Kadambot H. M. Siddique

**Affiliations:** ^1^Department of Chemical Engineering, Nadirshaw Eduljee Dinshaw (NED) University of Engineering and Technology, Karachi, Pakistan; ^2^Department of Polymer and Petrochemical Engineering, NED University of Engineering and Technology, Karachi, Pakistan; ^3^Department of Horticultural Sciences, Faculty of Agriculture and Environment, The Islamia University of Bahawalpur, Bahawalpur, Pakistan; ^4^Dr. Muhammad Ajmal Khan Institute of Sustainable Halophyte Utilization, University of Karachi, Karachi, Pakistan; ^5^Department of Environmental Sciences, Karakorum International University, Gilgit, Pakistan; ^6^Department of Botany, University of Karachi, Karachi, Pakistan; ^7^The UWA Institute of Agriculture, The University of Western Australia, Perth, WA, Australia

**Keywords:** bio-mediated, engineered nanomaterials (ENMs), agriculture, seed germination, plant growth, sustainable

## Abstract

The ever-increasing demand for agricultural food products, medicine, and other commercial sectors requires new technologies for agricultural practices and promoting the optimum utilization of natural resources. The application of engineered nanomaterials (ENMs) enhance the biomass production and yield of food crop while resisting harmful environmental stresses. Bio-mediated synthesis of ENMs are time-efficient, low-cost, environmentally friendly, green technology. The precedence of using a bio-mediated route over conventional precursors for ENM synthesis is non-toxic and readily available. It possesses many active agents that can facilitate the reduction and stabilization processes during nanoparticle formation. This review presents recent developments in bio-mediated ENMs and green synthesis techniques using plants, algae, fungi, and bacteria, including significant contributions to identifying major ENM applications in agriculture with potential impacts on sustainability, such as the role of different ENMs in agriculture and their impact on different plant species. The review also covers the advantages and disadvantages of different ENMs and potential future research in this field.

## Introduction

Growing food scarcity, declining land and water resources, and insufficient investment in sustainable agriculture are major factors limiting agricultural production. Climate change also adversely affects crop yields and is responsible for the deterioration of cultivable land and freshwater resources (Zulfiqar and Hancock, 2020; Hasnain et al., 2022). Therefore, the agriculture sector must focus on satisfying the tradeoff between the increasing demand for food crops and enhanced resource constraints by efficiently using land and water resources. Measures must be taken to prevent or minimize the adverse effects of climate change by adopting modern techniques such as using advanced materials in agricultural practices and promoting the smart utilization of natural resources. Numerous projects and investments are needed to fulfill the growing demand for agricultural products while adopting sustainable approaches (FAO, 2017).

Nanotechnology has many applications in daily life. The food and agriculture industry started adopting nanotechnology in different forms in 2003 (Xing et al., 2021). Research in this field has increased markedly in the last decade (Zulfiqar et al., 2019; Zulfiqar and Ashraf, 2021). Nanotechnology has various advantages, such as improved food quality and safety (Nandini et al., 2017). Nanotechnology enables scientists to engineer particles with unique, adaptive characteristics for various applications ranging from pharmaceuticals to electronics, fuel cells, water and gas treatments, and agriculture (Hochella Michael et al., 2019). Engineered nanomaterials (ENMs) are up to 100 nm in size and designed to have engineered physical and chemical properties with unique properties for various applications, such as sensors, thermoelectric materials, photocatalysts, dye-sensitized solar cells, nutraceuticals, drug delivery, biocidal agents, and nano-fertilizers (Bandala and Berli, 2019).

Some common types of ENMs are metallic oxides (e.g., Au, Ag, Fe_3_O_4_, TiO_2_, and ZnO), polymers, carbon ENMs (CENMs; e.g., fullerenes, carbon nanotubes, and graphene), hybrid particles, and polymer nanocomposites (Dontsova et al., 2019). Particle size and shape are the most important characteristics of ENMs, influencing their reactivity and transport mechanism and regulating the type and distribution of reactive sites of chemical reactions. Due to the high surface area to volume ratio, changes in surface structure impact the extent of nanomaterial reactivity in air, water, soil, or biota (Coman et al., 2019). Environmental factors of ENMs include, dissolution, evaporation, and aggregation (Hochella Michael et al., 2019).

In the last decade, researchers have been exploring advanced technologies, including nanotechnology, to fulfill population demands and address the global challenges in agriculture (FAO, 2017). Nanotechnology could be used to develop fertilizers and pesticides for crops (Ammar, 2018). Encapsulation of ENMs with other materials enhances plant nutrient uptake, consequently decreasing agrochemical waste. For example, nano-encapsulated materials have enhanced stability and solubility; thus, they can be used for efficient and controlled nutrient delivery (Shang et al., 2019). Nanocapsules act as a barrier preventing encapsulated ingredients from leaching into the land. when combined with other biological materials, may enhance plants’ tolerance to abiotic stresses like extreme temperatures and water deficiency Shang et al. (2019) which will be discussed later in detail. There is a clear need to develop ENMs and capitalize on their potential for increasing plant growth, resisting biocides, and minimizing environmental stresses such as flooding, salt, high temperatures, toxic metals, and drought that continue to increase with the increasing food demands and anthropogenic activities (Ali et al., 2017).

Global crop production is becoming insufficient due to lack of adequate nutrients and limitations of traditional agricultural methodologies that can easily be overcome by applying modern cultivation methods and using ENMs (Hao et al., 2018). ENMs are used extensively in modern plant technology and could play a major role in advanced biotechnological applications. Studies have shown that nanomaterials can support the transport of organic molecules into plant cells for nutrient uptake and disease prevention. Reactions such as particle dissolution, ion adsorption, cell interactions, uptake by microorganisms, and many other complex reactions affect the interaction of ENMs with organisms in various environmental media (Schwabe et al., 2014). Nano-agriculture is a branch of nanotechnology that deals with producing ENMs for application in pesticides, agrochemicals, water, soil improvers, and nano fertilizers (Shang et al., 2019). This review elaborates on and highlights the synthesis mechanisms of ENMs to understand the role of ENMs in agricultural systems. Ultimately, this review will help identify the major applications of ENMs in agriculture, potential impacts on sustainability, and the bio-mediated routes and materials for ENM synthesis. This review also highlights the application methodology and limitations of ENMs in the agricultural sector, knowledge gaps, and future trends in ENM development.

## Synthesis of engineered nanomaterials

**Figure 1:**
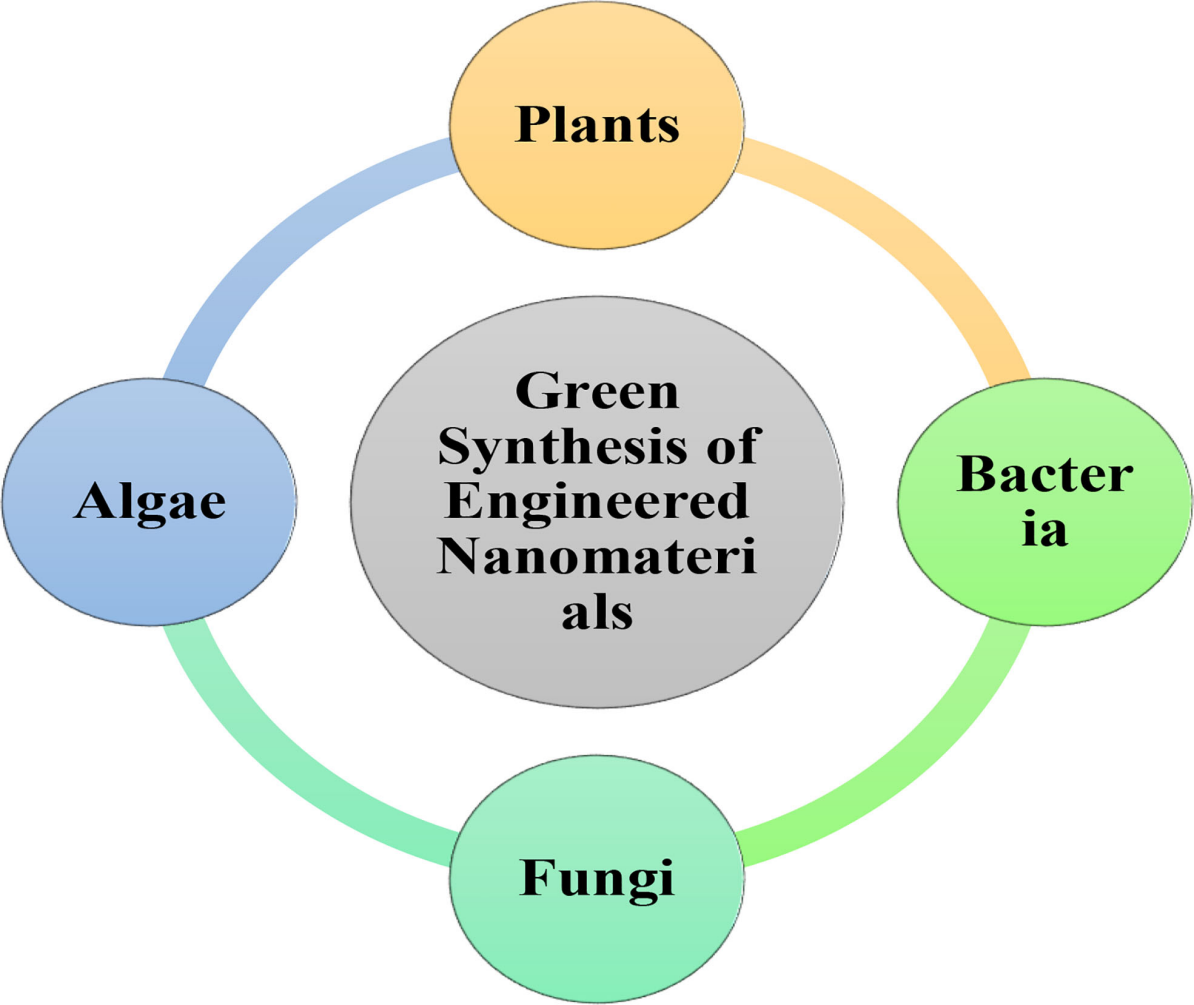
Biosynthesis routes of engineered nonmaterial by different organisms.

**Figure 2:**
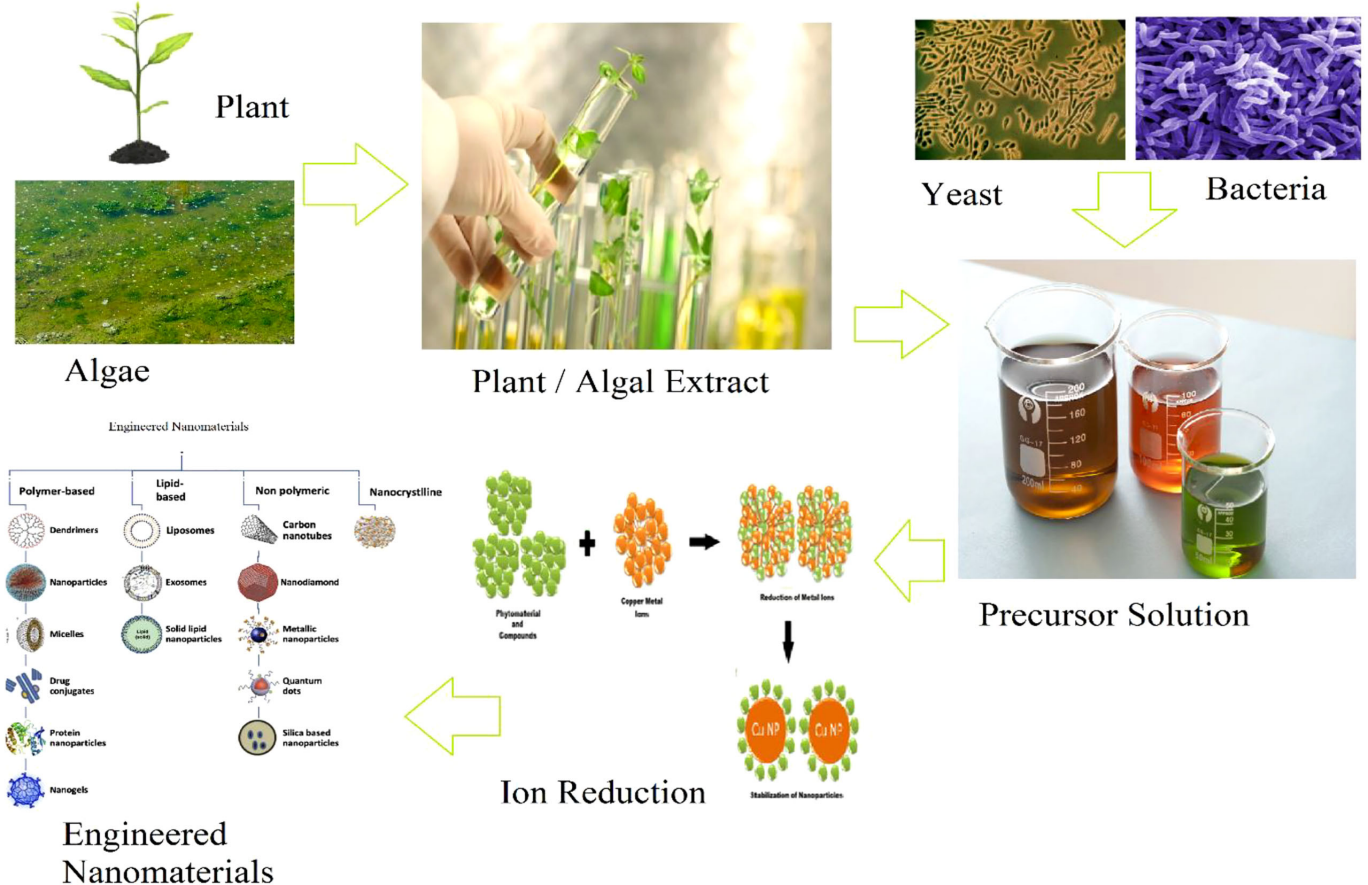
Green synthesis scheme of engineered nano materials from various resources.

Bio-mediated ENM synthesis is a time-efficient, low-cost, environmentally friendly, green technology. The green synthesis produces more stable ENMs than other means, and it is simple to scale up, and the risk of contamination is also lower. Plant leaves, as the site for photosynthesis, are food factories and can be used to synthesize NP, along with other plant parts, such as stems, seeds, bark, flowers, and roots (Kalpana and Devi Rajeswari, 2018). The precedence of using plant extract over conventional reducing and stabilizing agents such as sodium borohydride, sodium citrate, polyethylene glycol (PEG), polyvinylpyrrolidone (PVP), polyvinyl alcohol (PVA), Bovine serum albumin (BSA), Ethylene diamine tetra acetic acid (EDTA), Chitosan etc. for ENM synthesis is that plants are non-toxic, readily available, and possess many active agents that can facilitate the reduction and stabilization process during NP formation (Shafey, 2020). Most plant extracts contain organic micro- and macromolecules (e.g., hydroquinone, chlorophyll, estragole, allylbenzene, methylxanthine, dimethylxanthine, ascorbic acid, phenols, isoprenoid, poly-carbohydrates, flavonoids, alkaloids, proteins and other enzymes, amino acids, and alcohols), and act as reducing agents and capping ligands. This specific properties of organic micro- and macromolecules provide colloidal stability, prevent agglomeration/coagulation, and stop uncontrolled growth (Saglam et al., 2021). Other bio-mediated routes for ENM synthesis include microorganisms such as algae, yeasts, fungi, and bacteria that provide intra- or extracellular active sites for NP growth. These microreactors are more convenient for synthesizing ENMs in an economical and environmentally friendly manner than the other physical and chemical synthesis (Xu et al., 2021). **Figure 1** shows the bio-mediated synthesis routes which will be discussed in this section, including the effect these different synthesis routes will have on the characteristics and applicability of ENMs. **Figure 2** summarizes the general procedure of ENM synthesis of ENMs using green routes.

### Synthesis of engineered nanomaterials from plants

**Figure 3:**
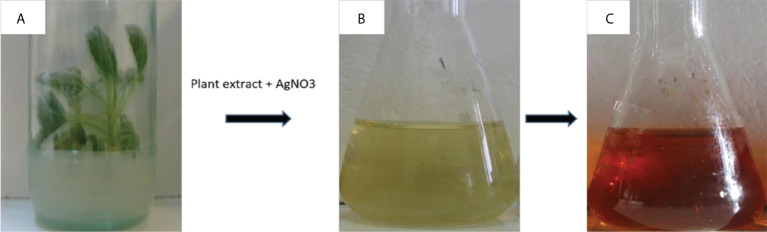
Color changes in the plant extract of *Salvia spinosa* after adding AgNO_3_ solution (Pirtarighat et al., 2019). **(A)** Plant leaves soaked in water. **(B)** Plant extract without AgNO3. **(C)** Plant extract with the addition of AgNO3. Copyright 2018.

Plant-mediated engineered nanomaterials (ENMs) synthesis is, low-cost, low-temperature, rapid, green technology using safe solvents (Chung et al., 2016; Munir et al., 2021). Palithya et al. (2021) used leaves of *Decaschistia crotonifolia* to synthesize silver (Ag) ENMs; combining the first leaf extract with silver nitrate (AgNO_3_) in a 1:2 ratio changed the solution color from yellowish light green to dark brown. The antimicrobial activity of the synthesized ENMs was analyzed against gram-positive *Staphylococcus aureus*, *Bacillus subtilis*, *Klebsiella pneumoniae* and *Escherichia coli*. The biosynthesized ENMs exhibited better antibacterial activity against all bacterial species; while moderate efficacy compared to the standard antibiotic *Amoxyclavhr*. These ENMs had optimum catalytic activity to enhance the dye degradation rate by rapid reduction of nitrophenol to aminophenol. Hence, these ENMs could be used in various pharmaceutical and industrial applications. Hemlata et al. (2020) used leaf extract of *Cucumis prophetarum* to synthesize silver (Ag) ENMs. The plant extract was mixed in a 1:9 ratio with 0.001 M AgNO_3_ solution. The mixture was heated at 80°C. The precipitates were then centrifuged and dried to obtain brown-colored silver (Ag) ENMs. The antimicrobial activity of the green-synthesized Ag ENMs and aqueous leaf extract of *C. prophetarum* was analyzed against Gram-positive *Staphylococcus aureus* and Gram-negative *S. typhi*; the ENMs showed greater antibacterial activity against Gram-negative than Gram-positive bacteria. The antiproliferative activity of the green-synthesized Ag-ENMs against cancer cell lines was also tested, with cell viability gradually decreasing with increasing concentration of Ag-ENMs. **Figure 3** shows ENM synthesis using *Salvia spinosa*.

**Figure 4:**
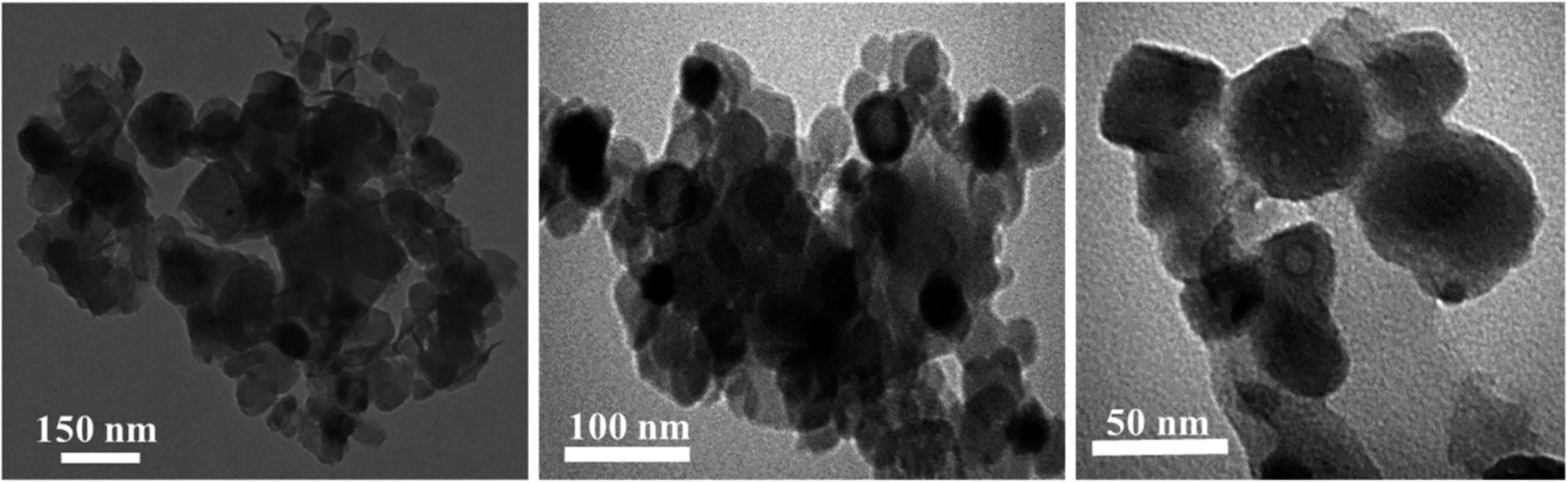
TEM images of copper oxide ENMs synthesized by lavender and green tea leaf powders using the solid-state method (Khaldari et al., 2021). Copyright 2021.

Khaldari et al. (2021) synthesized copper (II) oxide (CuO) ENMs using lavender and green tea leaves. Copper sulfate (CuSO_4_.5H_2_O) and lavender (or green tea) powder were mixed in varying proportions, with the resulting mixture ground into a homogeneous powder, heated at 600°C for 4 hours, and then cooled to room temperature before suspending the residue in ethanol and centrifuging to obtain the required ENMs. **Figure 4** shows scanning electron microscopy (SEM) images of the nanomaterials. The X-ray diffraction (XRD) of green synthesized CuOENMs revealed that increasing the plant powder increased the purity of ENMs, and lavender powder produced more pure CuO ENMs than green tea leaf. Compared with chemically synthesized CuO ENMs, the biosynthesized ENMs had a less inhibitory effect on seed germination. Also, green and chemically synthesized CuO ENMs at low concentrations (i.e., 4 μg mL^–1^) increased root and shoot lengths of lettuce and tomato seedlings (Khaldari et al., 2021).

*Abutilon indicum* leaf extract was used to synthesize CuO ENMs using the green combustion method (Ijaz et al., 2017). A homogeneous solution of copper (II) nitrate trihydrate and 0.3 g *Abutilon indicum* was prepared in 20 mL distilled water, heated at 400°C in a muffle furnace to form CuO ENMs, which were filtered and washed to remove impurities. The XRD results of the synthesized CuO ENMs revealed a highly crystalline structure due to the leaf extract, which controls particle size. Furthermore, the antimicrobial activity of the CuO the green ENMs was analyzed against *S. aureus, Klebsiella*, and *B. subtilis* by measuring the radius of inhibition zones, it was observed that the highest inhibition was observed for *B. subtilis* with 15 mm inhibition zone. When compared with the standard drug ampicillin, the CuO ENMs exhibited strong antimicrobial activity with a minimum inhibitory concentration (MIC) of 0.05 mg mL^–1^, revealing that the CuO ENMs released copper (Cu) ions which penetrated the bacterial cell membrane, damaging its formation by attaching to negatively charged cell walls.

Ahmad et al. (2021) synthesized iron oxide ENMs using *E. hirta* leaf. The leaf extract was combined with 0.1 M ferrous sulfate heptahydrate and 0.1 M ferric chloride at constant pH 9 using standard sodium hydroxide (NaOH) solution; brown-colored iron-oxide ENMs formed after 3 h, which were centrifuged to remove impurities. The antimicrobial activities of the engineered ENMs were analyzed by quantifying the size inhibition zone on agar plates and determined to be directly proportional to their concentration. Erci and Cakir-Koc (2021) synthesized iron oxide ENMs (FeO) using *T. spicata*. The solution was formed by mixing *T. spicata* in 100 mL water, heated at 60°C for 30 min, and filtered. The extract was mixed with 0.1 M ferrous sulfate (FeSO_4_.7H_2_O) to synthesize iron oxide nanomaterials. The antimicrobial activity of the FeO ENMs was analyzed for Gram-positive and Gram-negative bacterial species. *B. cereus* had MIC and minimum bactericidal concentration (MBC) values of 200 mg mL^–1^, and *S. typhimurium* had the lowest MIC of 100 mg mL^–1^. The inhibition percentage was directly proportional to the fraction of leaf extract used to synthesize ENMs. The cytotoxicity of FeO ENMs on L929 cells was less than that for FeSO_4_.7H_2_O. **Table 1** lists more examples of ENM synthesis using plant extracts

**Table 1:** Applications of Engineered Nano materials in seed germination.

Engineered Nanomaterials(ENMs)	Plants	Size (nm)	Shape	Antimicrobial applications	References
Ag-NP	*Impatiens balsamina* and *Lantana camara*	3–20	Spherical	*Escherichia coli*	(Aritonang et al., 2019)
	*Salvia spinosa*	5.13	Spherical/ovl	*Bacillus subtilis, Bacillus vallismortis*, and *Escherichia coli*	(Pirtarighat et al., 2019)
	*Azadirachta indica*	34	Spherical	*Escherichia coli* and Staphylococcus aureus	(Ahmed et al., 2016)
	*Ocimum sanctum*	10 ± 2 (synthesized from roots); 5 ± 1.5 (synthesized from stems)	Spherical	–	(Ahmad et al., 2010)
	*Cannabis sativa* (hemp)	200–300	Spherical	*Staphylococcus aureus* and *Escherichia coli*	(Mandal et al., 2021)
Zn-NP	Chamomile (*Matricaria chamomilla* L.) flower, olive (*Olea europaea*) leaf, and red tomato (*Lycopersicon esculentum* M.) fruit	41.0 ± 2.0 (*Olea europaea*) 51.2 ± 3.2 (*Matricaria chamomilla*) 51.6 ± 3.6 (*Lycopersicon esculentum*)	Cubic	*Xanthomonas oryzae* pv. *oryzae* (Xoo) strain	(Ogunyemi et al., 2019)
	*Ocimum americanum*	21	Spherical	*Bacillus cereus* *Klebsiella pneumonia* *Staphylococcus aureus* *Vibrio parahaemolyticus* *Escherichia coli* *Pseudomonas aeruginosa* *Salmonella typhi* *Xanthomonas citri* *Candida albicans* and *Aspergillus parasiticus*	(Narendra Kumar et al., 2019)
	*Cinnamomum tamala*	26.57	Spherical and hexagonal	*Staphylococcus aureus*	(Agarwal et al., 2019)
	*Stevia rebaudiana*	8.35	Spherical	*-*	(Alijani et al., 2019)
	*Azadirachta indicia*	19.57 ± 1.56	Non-spherical	Pneumococci strains	(Sohail et al., 2020)
	*Brassicaceae*	100–150	Spherical	*Bacillus subtilis, S. aureus, Salmonella typhimurium, Aspergillus niger, Fusarium oxysporum*, and *Penicillium digitatum*	(Perveen et al., 2020)
	*Caryophyllus aromaticus*	18	Oval and spherical	*Acinetobacter baumannii*	(Hou et al., 2020)
	*Carica papaya*			*Sclerotinia sclerotiorum, Rosellinia necatrix* and *Fusarium* species	(Dulta et al., 2021)
Cu-NP	*Caesalpinia bonducella*			*Staphylococcus aureus* and *Aeromonas*	(Sukumar et al., 2020)
	*Catha edulis*	13.07	Rice-grain-shaped	*Staphylococcus aureus, Streptococcus pyogen, Klebseilla pneumonia, Escherichia coli*	(Sukumar et al., 2020)
	Walnut leaf extract	80	Spherical and crystalline	*Escherichia coli*	(Sukumar et al., 2020)
	*Adhatoda vasica*	7–11	Composite flakes	Candida albicans and the bacteria Klebsiella pneumoniae	(Bhavyasree and Xavier, 2020)
	*Ocimum basilicum*	70	Spherical	*Escherichia coli* and *Staphylococcus aureus*	(Altikatoglu et al., 2017)
	Herbal tea (*Stachys lavandulifolia*) flower extract	20–35	Spherical	Catalytic activity	(Veisi et al., 2021)
	*Hagenia abyssinica* (Brace)	34.76	Spherical, hexagonal, triangular, cylindrical and irregularly shaped	*E. coli, Pseudomonas aeruginosa, Staphylococcus aureus*, and *Bacillus subtilis*	(Murthy et al., 2020)
	*Gloriosa superba* L.	5–10	Spherical	*Klebsiella aerogenes, Pseudomonas desmolyticum*, and *Escherichia coli, S. aureus*	(Naika et al., 2015)
Fe-NP	*Bauhinia tomentosa*	70	Face-centered cubic	Synthesis of 1,3-diolein	(Lakshminarayanan et al., 2021)
	*Carica papaya*	21.59	Non-uniform	*Klebsiella* spp., *Escherichia coli*, *Pseudomonas* spp., *S. aureus*	(Bhuiyan et al., 2020)
	Green tea	50–80	Spherical	Removal of hexavalent chromium	(Hao et al., 2021)
	*Platanus orientalis*	78–80	Spherical	*Aspergillus niger* and *Mucor piriformis*	(Devi et al., 2019)
	Peanut skin extract	10.6	Spherical	Chromium removal	(Pan et al., 2020)
	*Moringa oleifera*	138–475	Spherical	*Escherichia coli*	(Katata-Seru et al., 2018)

### Synthesis of engineered nanomaterials from algae

**Figure 5:**
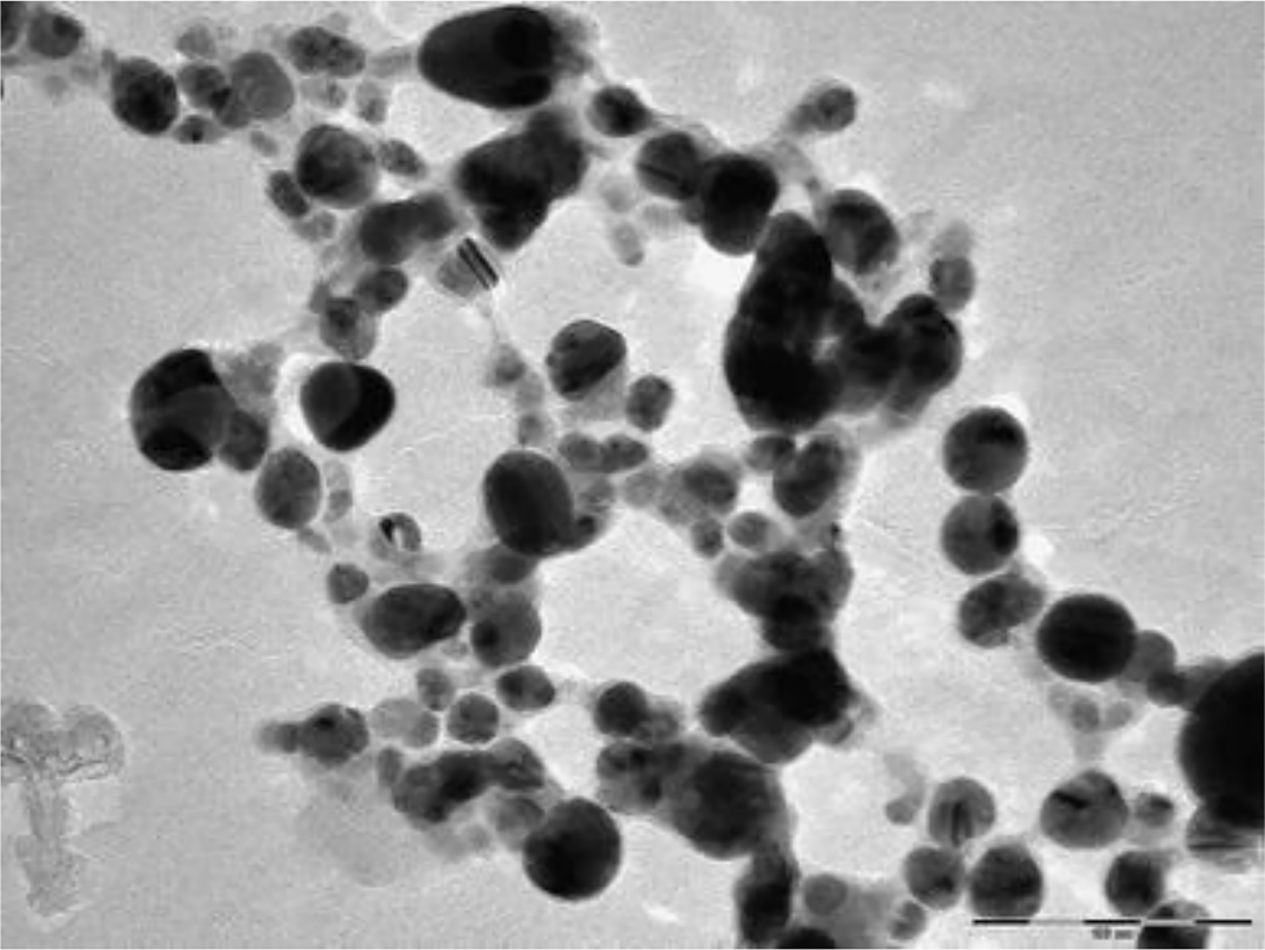
TEM image of copper ENMs (Engineered Nanomaterials) synthesized from algal extract of *Bifurcaria bifurcata* (Abboud et al., 2014). Copyright 2013.

Algae are readily available and eco-friendly, with rich source of metabolites and optimum metal uptake capacity for use as reducing agents for nanoparticle synthesis (Abideen et al., 2022; Hasnain et al., 2022). In addition, algae accumulate higher lipids and potential source of biodiesel using saline lands and brackish water. Aboelfetoh et al. (2017) synthesized silver ENMs using *Caulerpa serrulata* from the Red Sea coast, Egypt. First, the algae were powdered after cleaning and natural drying; 1 g algae powder was cooked in 100 mL water for 10 min at 70°C before mixing 5–25 mL *C. serrulata* extract with 10^−3^ M AgNO_3_ and adjusting the total volume to 100 mL. The formation of ENMs was depicted by yellow to brown color depending on the algal extract used, reaction time, temperature, and pH of the solution. The catalytic activity of the Ag-NP was evaluated in a reducing aqueous solution of Congo Red dye in the presence of sodium borohydride (NaBH_4_) at room temperature. It was observed that without Ag ENMs no dye degradation was observed even after 3 hours, once biosynthesized Ag ENMs have added dye degradation started within 5 minutes. The antibacterial activity of the Ag-NP increased with increasing concentration of Ag-NP. The highest inhibition tendency occurred for *E. coli*, and minimum inhibition occurred for *S. typhi*. Dhas et al. (2013) synthesized silver chloride (AgCl) ENMs from *Sargassum plagiophyllum*. The seaweed was washed to remove impurities and dried in the shade for 8-10 days before grinding to a powder. A seaweed extract was prepared by mixing 5 g seaweed powder with 50 mL water. To synthesize AgCl ENMs, varying amounts of seaweed extracts (50-500 × 10^–6^ L) were mixed with 5 mL of 0.001 M AgNO_3_ solution and kept at room temperature for 1 day. The AgCl ENMs were depicted as yellowish-brown, spherical shapes (18-42 nm) in TEM images. The antibacterial activity of the AgCl ENMs was investigated against *E. coli*. The inhibitory action proportional to the quantity of AgCl ENMs, as depicted by larger inhibition zones was observed. Abboud et al. (2014) used *Bifurcaria bifurcata* (brown algae) to synthesize copper ENMs. The algae were washed with water (until it neutralized to pH 7), dried for 12 h at 60°C), and ground to a powder. The powder was boiled in water (1:5 ratio) for ~1 h, mixed with 0.001 M copper sulfate solution (1:10 ratio), and heated at about 100°C under continuous stirring to obtain dark brick red colored precipitates. The formation of spherical copper oxide ENMs (5-45 nm) was depicted in TEM images, with a few elongated ENMs (**Figure 5**). The antimicrobial activity of the copper oxide ENMs was considerable for Gram-negative and Gram-positive bacterial species (*E. aerogenes* and *S. aureus)* compared to the algae extract which had no antimicrobial activity.

Eroglu et al. (2013) synthesized spherical palladium nanocrystals from green microalgae (*Chlorella vulgaris*) using aqueous sodium tetrachloro-palladate Na_2_ (PdCl_4_) photosynthetic reactions. The nanocrystals were embedded in electrospun chitosan mats as a catalyst for the Mizoroki–Heck cross-coupling reaction. Cross-linked chitosan mats (3 × 2 cm rectangular shape) developed by electrospinning were dipped into a one-month-old mature microalgal culture in 25 mg L^–1^ Na_2_ (PdCl_4_). The control sample comprised chitosan mats treated with 25 mg L^–1^ Na_2_ (PdCl_4_) solution without microalgae. The efficacy of the biogenic nano palladium-chitosan mats was analyzed as catalyst support in the Mizoroki-Heck reaction. Six dried catalyst mats were placed into a solution containing iodobenzene, butyl acrylate, triethylamine and dimethylformamide (DMF) at 80°C for 16 h; after each reaction, the catalyst was recovered for regenerative studies after washing with DMF under nitrogen gas to prevent oxidation that may lead to the reproduction of Pd (0) ENMs. The catalyst was recyclable for a minimum of four reactions, with reaction yields of 68, 62, 45, and 36% (by weight) for the first to fourth cycles, respectively, much higher than the control at only 5%.

Ishwarya et al. (2018) synthesized zinc oxide (ZnO) ENMs using *Ulva lactuca* marine seaweeds. The seaweed was washed with water, cut, and dried in the air. A 10% solution of seaweed powder (5 g powder in 50 mL water) was obtained after boiling for 20 min. The extract was filtered and stored for later use when 5 mL extract was mixed with a 95 mL aqueous solution of 1 mM zinc acetate at 70°C with continuous stirring for 3-4 h. After centrifugation at 4,000 rpm for 10 min, yellowish-white precipitates were collected and washed with water before heating at 450°C for 4 h. Agglomerates of sample sponge-like asymmetrical shaped ENMs (10-50 nm) were depicted in TEM images. The inhibition percentage of antibacterial activity of ZnO ENMs against Gram-positive and Gram-negative species was observed between 80 to 95% in dark and visible light; specifically, *lactuca*-fabricated ZnO ENMs had an 82% reduction rate for *B. licheniformis*, 84% for *B. pumilus*, 80% for *E. coli*, and 79% for *P. vulgaris* (79%). Furthermore, the highest inhibition percentage of 100% occurred for *A. aegypti* in 50-μg mL^–1^ ZnO ENMs at 24 h.

### Synthesis of engineered nano materials from fungi

Fungi include many multicellular and unicellular species that used in various processes since early civilization. They play an important role in ecosystems, regulating the never-ending cycle of greenhouse gas emissions. Fungi have positive and negative impacts on our daily lives (Hussin, 2018). They are used in many industrial processes, including manufacturing antibiotics, steroids, and hormones (e.g., penicillin and cephalosporin), fermentation processes, organic acids (e.g., citric acid), and protein and vitamin production. While fungi cause many plant diseases for crops, several species improve soil quality and health, helping plants cope with saline and drought stresses (Ramasamy, 2020). Many fungal species used as protein-rich foods, including some mushrooms, morels, and truffles. Fungi can decompose all materials except plastics. Various fungal species are present in forest soils, withstanding stresses such as changing pH, decomposing cellulose lignin and other organic residues, and importing nutrients through hyphae for plant uptake of nitrogen, phosphorus, micronutrients, and water (Morris, 2004). Due to their rapid growth, simple synthesis, and short life cycle, fungi used in microbiology, biochemistry, and genetic engineering labs to study various metabolisms (e.g., the ‘one gene, one enzyme’ theory in *Neurospora* for which George W Beadle and Edward L Tatum won the Nobel Prize for Physiology or Medicine in 1958).

Yeasts are single-celled fungi widely used in various fermentation processes. The synthesis of ENMs through yeast simplifies the fermentation process, at ambient temperature; it is also very low cost and environmentally friendly. Moreover, synthesis through yeast has an advantage over other microbial species because yeast is the highest industrially produced microorganism in the world for different fermentation-based products, such as wine and bread. Bao et al. (2010) synthesized cadmium telluride quantum dots using yeast cells grown on a modified Czapek’s medium (450 g sucrose, 15 g sodium nitrate (NaNO_3_), 5.0 g potassium hydrogen phosphate (K_2_HPO_4_), 2.5 g potassium chloride (KCl), 2.5 g magnesium sulfate (MgSO_4_.7H_2_O), and 30.0 g sodium citrate tribasic dihydrate dissolved in 5 L deionized water) and incubated for 2 days at 35°C. The yeast culture was combined with 400 mL cadmium chloride (CdCl_2_) (0.04 mol L^–1^), and 100 mL sodium tellurite (Na_2_TeO_3_) (0.04 mol L^–1^), 7.5 g MSA, and 5.0 g NaBH_4_, and incubated in a water bath. The final product comprised well-dispersed crystalline CdTe ENMs in a cubic zinc-blended shape (3.6 nm) after centrifugation dialysis using a dialysis membrane to remove residual chemicals and yeast cells.

Fernández et al. (2016) synthesized Ag ENMs using *Cryptococcus laurentii* and *Rhodotorula glutinis* incubated at 28°C and 100 rpm for 24 h in Muller–Hinton broth (MHB), containing 2 g beef infusion, 17.5 g acid casein peptone, and 1.5 g corn starch at neutral pH, with 2.0×10^6^ yeast cells in suspension and centrifuged before further use. The Ag ENMs were synthesized by adding 1 mL of 1 mM aqueous silver nitrate solution in 100 mL of yeast culture and stirring at 100 rpm at 28°C for 48 h in the dark. Particle formation (15-220 nm) was validated by the solution changing from yellow to deep brown using TEM images. Many fungi species rot fruits and vegetables and ultimately reduce their shelf life; therefore, the inhibition ability of biosynthesized Ag ENMs was assessed for *B. cinerea, P.expansum, A. niger, Alternaria* sp., and *Rhizopus* sp; the synthesized Ag ENMs from *R. glutinis* had higher antimicrobial activity than from *C. laurentii*. At 3 ppm, the ENMs synthesized from *R. glutinis* had antifungal efficacy comparable to antifungal agent iprodione against all fungi species in question (except *Rhizopus*). Eugenio et al. (2016) synthesized silver and silver chloride using yeasts produced from the gut of ten termites (*Cornitermes cumulans*). The gastrointestinal matter was placed on *Sabouraud* agar plates and incubated at 28°C for 48 h. For the incubation of separate clones, the colonies were re-incubated after dilution. Fifteen of these separate clones were used to synthesize AgCl ENMs; each isolated plate was inoculated in a liquid medium containing 4% glucose, 1% peptone, and 1% yeast extract at pH 6.5 at 30°C for 24 h. The media was mixed with 3.5 M silver nitrate solution and further incubated at 30°C in the dark with constant stirring at 150 rpm for 7 days. The silver chloride ENMs were separated from the mixture by centrifugation to obtain circular ENMs (2–10 nm), as confirmed by TEM images. To analyze the cell inhibition efficacy of biosynthesized silver chloride ENMs against *S. aureus* and *K. pneumoniae*, the ENMs were suspended in stabilizing solution comprising 1% sodium citrate with a basic pH 8 for 24 h. A dose-dependent inhibition effect was observed against both bacterial species at a maximum loading of 50μg mL^–1^ (83% and 85% against *S. aureus* and *K. pneumonia*, respectively).

Tian et al. (2010) synthesized mesoporous zirconium phosphate using yeast grown in 100 mL of aqueous glucose solution (2 wt.%) at room temperature for 0.5 h before adding 3.2 g zirconyl chloride (ZrOCl_2_.8H_2_O). After 12 h, 100 mL of 0.2 M disodium hydrogen phosphate was mixed into the solution, maintaining the pH at 1-3 using 0.05 M HCl. After 2 h of mixing, a white gelatinous texture formed; after 48 h of storage, the mixture was centrifuged and washed vigorously with water and then ethanol. The product was dried at 80°C for 24 h to obtain a wormhole-like mesoporous structure from agglomerates of zirconium phosphate ENMs, which were analyzed for their efficiency as air electrodes for electro-catalytic activity in the oxygen reduction reaction (ORR) in alkaline solution. Due to its open mesoporous structure that can enhance mass transfer and neutralize polarization, this process had superior results to electrolytic manganese dioxide (EMD) air electrodes. These results showed that these mesoporous particles could be used for fuel cell applications.

### Synthesis of engineered nanomaterials from bacteria

Kanmani and Lim (2013) synthesized silver ENMs using a lactic acid bacterium that acts as a reducing and stabilizing agent. First, exopolysaccharide (EPS) was produced using bacterium: *Lactobacillus rhamnosus* GG was incubated in 1 L MRS (De Man, Rogosa, and Sharpe) agar broth at 37°C for 18 h and then heated at 100°C for 15 min to deactivate EPS-degrading enzymes before centrifuging to remove probiotic cells and debris. The supernatant was mixed with ethanol (95%) and cooled at 4°C for 12 h. The EPS precipitates were collected and washed with distilled water before mixing 20 mL EPS with 9 mM AgNO_3_. The mixture was stored for 60 days in the dark; a 10 h incubation resulted in a yellowish solution, indicating the formation of silver ENMs, with sizes ranging from 2-15 nm and shapes ranging from the spherical, hexagonal, rod, and triangular. The antimicrobial activity of biogenic silver ENMs was dose-dependent against *E. coli, L. monocytogenes* (food-borne pathogen), and multi-drug resistant pathogens *K. pneumonia* and *P. aeruginosa*. *P. aeruginosa* had the highest inhibition zones, while the growth of *L. monocytogenes* was only inhibited at high nanoparticle doses (i.e., 2 mg mL^–1^). Likewise, the efficacy of synthesized ENMs was dose-dependent against fungal pathogens *Aspergillus* spp. and *Penicillium* spp., particularly for *Aspergillus* spp. He et al. (2007) synthesized gold ENMs using the photosynthetic bacterial species *Rhodo-pseudomonas capsulate* by first maturing in a growth medium comprising pyruvate yeast extract, sodium chloride, ammonium chloride, and potassium hydrogen phosphate at pH 7 at 30°C for 3 days and then centrifuging to remove residual impurities. After washing with water, 1 g of the bacteria was mixed in 20 mL of 1×10^−3^ M chloroauric acid, neutralizing the pH with 0.1 M NaOH and 0.1 M HCl. As confirmed by TEM images, UV-visible spectra confirmed the nanoparticle synthesis with spherical ENMs (10–20 nm). Hence, synthesis of ENMs using microbial species is a time-efficient, ecofriendly, simple process that produces particles of uniform shape and size. **Table 2** summarizes research studies using microbes to synthesize ENMs.

**Table 2:** Examples of synthesis of engineered nanomaterials using algae/bacteria/yeast.

Nanoparticle (NP)	Plant species	Size (nm)	Shape	Applications	References
Ag-NP	*Padina pavonia*	49.58–86.37	Spherical, triangular, rectangular, polyhedral, and hexagonal	–	(Abdel-Raouf et al., 2019)
	*Trichodesmium erythraeum*	26.5	Cubic	*Staphylococcus aureus, Proteus mirabilis*, *Escherichia coli*, *Staphylococcus aureus* and *Streptococcus pneumoniae*	(Sathishkumar et al., 2019)
	*Caulerpa racemosa*	5–25	Face-centered cubic	*Staphylococcus aureus and Proteus mirabilis*	(Kathiraven et al., 2015)
	*Botryococcus braunii*	40–100	Face-centered cubic	*Pseudomonas aeruginosa and E. coli, Klebsiella pneumoniae*, *S. aureus* and *Fusarium oxysporum*	(Arya et al., 2018)
	*C. racemosa*	5–25	Face-centered cubic	*S. aureus and Proteus mirabilis*	(Kathiraven et al., 2015)
	*Cyanobacterium Oscillatoria limnetica*	3.30–17.97	Quasi-spherical	*E. coli* and *Bacillus cereus*	(Hamouda et al., 2019)
	*Gracilaria birdiae*	20.2-94.9	Spherical	*E. coli* and *S. aureus*	(de Aragão et al., 2019)
Pd-NP	*Sargassum bovinum*	50–150	Spherical	Electrocatalytic activities toward hydrogen peroxide	(Siddiqi and Husen, 2016)
	*Spirulina platensis*	10–20	Spherical	Tested as adsorbent	(Sayadi et al., 2018)
Cu-NP	*Macrocystis pyrifera*	2–50	Spherical		(Araya-Castro et al., 2021)
	*Eryngium caucasicum*	<40	Spherical	*E. coli, Salmonella typhimurium, Bacillus cereus, and S. aureus*	(Hasheminya and Dehghannya, 2020)
Zn-NP	*Chlorella *	20 ± 2.2	Hexagonal	Photodegradation of Dibenzothiophene (DBT) aqueous solution	(Hasheminya and Dehghannya, 2020)
	*Ulva lactuca*, and *Stoechospermum marginatum*	12–17	Spherical and round	Antibacterial, antifungal, and anticancer applications	(Anjali et al., 2021)

## Applications of engineered nanomaterials in agriculture

In recent years, nanomaterials have been studied for various applications ranging from energy generation to food production. For these applications, nanomaterials are engineered to impart application-specific properties. For example, in agriculture, interactions of ENMs with food and non-food crops through various routes have been studied to enhance production and yield. ENMs are mostly applied to plants through roots (as nano-fertilizers) or leaves (by foliar spraying). The physiological mechanisms of leaves and roots are different. Consequently, the absorption mechanisms of ENMs are also different through these organs. Roots release exudates that inhibit the absorption of ENMs, while leaves can isolate ENMs in the cell wall after spraying on leaves. Foliar applied nutrients are transported downwards and further distributed to other parts of the plant (Kannan and Charnel, 1986).

**Figure 6:**
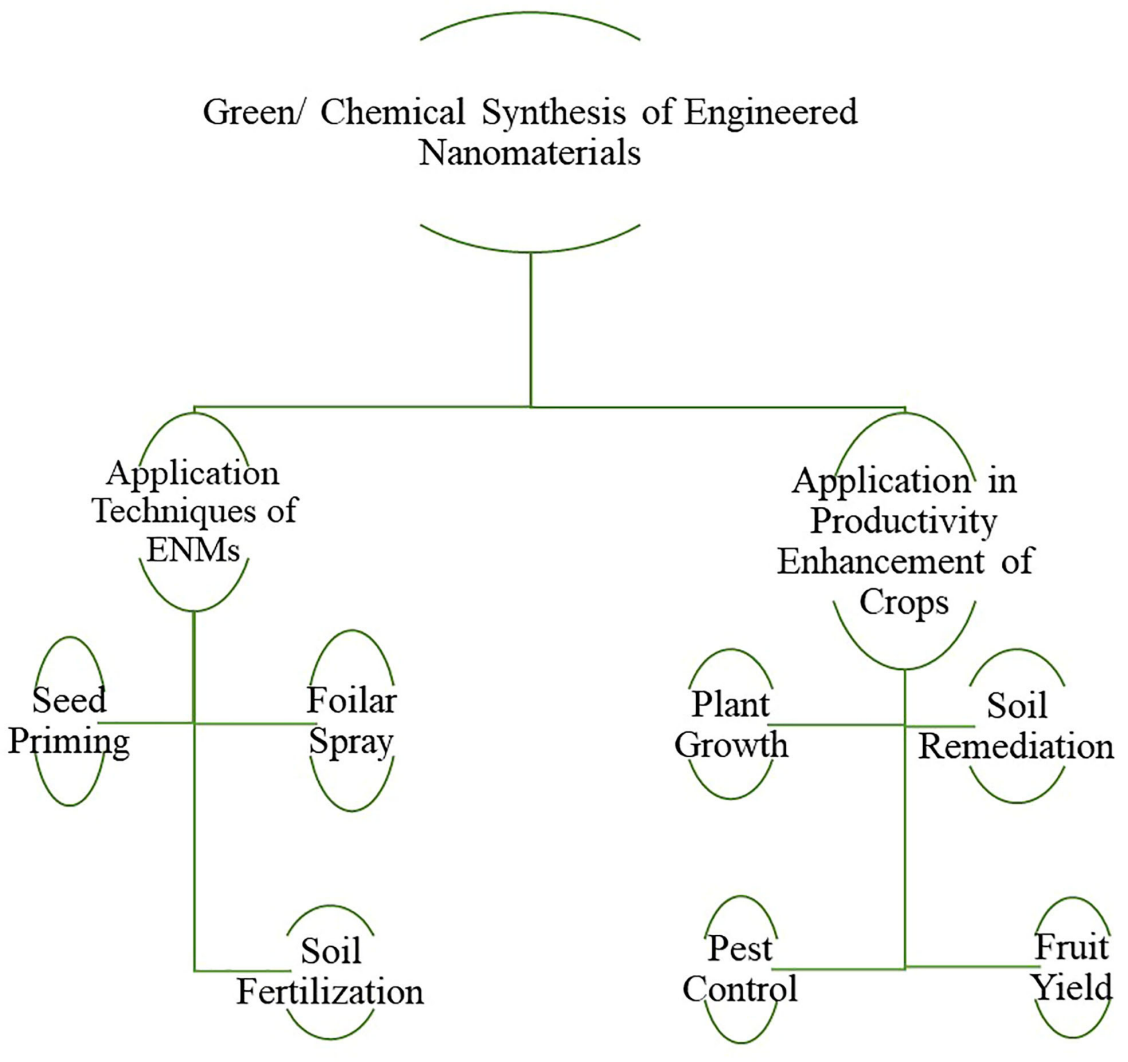
Application of engineered nanomaterials in agricultural productivity.

Similarly, ENMs sprayed on the leaves increased ENMs content in the root system, which may help root cells absorb nutrients and improve their disease resistance. Compared with soil fertilization, foliar application of nano fertilizers can compensate for nutrient deficiency in plants more quickly by accumulating the applied nutrient (Alshaal and El-Ramady, 2017). Zinc ENMs were evaluated as nano fertilizers using soil fertilization technique for enhancing the growth of pearl millet. Positive effects were observed on the shoot and root lengths, chlorophyll content, total soluble leaf protein, and consequently grain yield of 37.7% as compared to the control plant i.e. without nano Zinc ENMs (Tarafdar et al., 2014). Foliar application of Zinc ENMs substantially increased plant growth and dry biomass (Najafi Vafa et al., 2015). Also, foliar sprays of TiO_2_ increased plant total dry matter by enhancing N assimilation, photo-reduction activities of photosystem II and electron transport chain and scavenging the reactive oxygen species (Raliya et al., 2015). The chlorophyll, carotenoids, and anthocyanin contents of maize crops increased significantly after foliar application of TiO2, increasing the yield (Morteza et al., 2013). Foliar application of ENMs, which has many advantages not available in traditional methods, is a research direction with great potential in the agro-industry. **Figure 6** elaborates on the ENMs application techniques and impact on enhancing plant productivity.

### Seed germination applications

Seed germination and seedling growth are the backbones of crop yield and productivity (Zulfiqar, 2021). Seed priming is a technique used to improve the quality of seeds and their tolerance to environmental stresses. Priming seeds with nanomaterials is a novel method to increase germination rates and decrease seed aging (Itroutwar et al., 2020). The effect of bulk zinc acetate and algal synthesized zinc oxide (ZnO) ENMs on maize seed germination and plant growth. Maize seeds primed with varying concentrations of ZnO ENMs significantly improved the percentage of early seed germination compared to bulk zinc acetate and hydro primed (control) seeds. The 100 mg L^–1^ ZnO treatment produced the highest germination percentage (87%) after one-week and the highest root and shoot lengths and widths. Panda (2017) soaked rice (*Oryza sativa* L.) in zinc nanoparticle solutions (5-50 mg L^–1^) for 1 h under continuous stirring in an incubator. Seeds primed with Zn particles had longer radicles and plumules, higher radical and plumule fresh and dry weights, and higher relative water contents (RWC) of radices and plumules than untreated seeds. Mohammed et al. (2019) observed the effect of silica ENMs (SiO_2_) at varying concentrations (100-300 mg L^–1^) on the growth parameters of cucumber seeds. At 200 and 300 mg L^–1^, silica-primed seeds reached 100% germination in 10 days. Seed priming also decreased the mean germination time from 4.1 days (no priming) to 2.8 days (200 mg L^–1^). Siddiqui and Al-Whaibi (2014) examined the effect of SiO_2_ ENMs (0–10 g L^–1^) on the germination characteristics of tomato seeds (*L. esculentum* Mill. cv. Super Strain B). Seed germination increased with increasing SiO_2_ concentration, peaking at 8 g L^–1^.

Baz et al. (2020) observed the effect of carbon ENMs (CENMs) under saline conditions on the seed germination characteristics of different lettuce species. Lettuce seeds were primed with a 0.3% (wt) solution of CENMs (90–110 nm) for 2–14 h, followed by washing with water and then salt solution before incubating for 9 h. For the salt treatment, seeds were primed with 150-250 mM NaCl for 4 h. The CENMs pretreatment significantly reduced the adverse effects of saline and hot conditions on seed germination. Even 2 h of CENMs priming improved seed resistance to saline, with germination rates reaching 100% compared to only 10% under saline conditions without no CENMs treatment also the germination rates of seeds under thermal stress of 34°C were observed to enhance in seeds primed with CENMs up to 90% as compared to 405 without CENMs. Only a few species had very low germination rates, even after priming for 10 h. **Table 3** lists some studies using ENMs for seed priming and germination.

**Table 3:** Applications of Engineered Nano materials in seed germination.

Nanoparticle (NP)	Plant Species	Parameters analyzed	References
Ag-NP	*Brassica juncea* and *Vigna radiata*	Seed germination, seedling growth, and chlorophyll studies	(Khedkar and Khedkar, 2020)
	Lentil seeds	Germination index, root and shoot lengths, and fresh and dry weights	(Hojjat and Hojjat, 2016)
	*Satureja hortensis*	Seedling weight and length and germination rate	(Nejatzadeh, 2021)
	Fenugreek seeds	Germination rate, root and shoot length, fresh and dry mass	(Hojjat and Kamyab, 2017)
Zn-NP	Lettuce seeds	Germination rate and biomass	(Rawashdeh et al., 2020)
	*Allium cepa* L.	Seed germination and seedling growth	(Tymoszuk and Wojnarowicz, 2020)
	Peanut seeds	Seed germination rate, seedling vigor, plant growth rate, and chlorophyll content	(Prasad et al., 2012)
	Onion	Number of seeded fruits and seed yield per umbel	(Lawre and Raskar, 2014)
	Chickpea seedlings	Shoot dry weight	(Burman et al., 2013)
Si-NP	*Thymus kotschyanus*	Seed germination, early growth traits, percent and rate of germination, root and shoot lengths, seedling fresh and dry weights, and seed vigor index	(Khalaki et al., 2016)
	*Lens culinaris*	Seed germination, vigor index, and biomass	(Khan and Ansari, 2018)
	*Vicia faba* L.	Germination and seed growth	(Roohizadeh et al., 2015)
	*Arabidopsis thaliana*	Germination rate and seed growth	(Crişan et al., 2009)
Carbon nanotubes	*Lycopersicum esculentum* Mill., *Allium cepa, Brassica rapa* and *Raphanus sativus*	Germination percentage and rate, seedling length, seedling fresh and dry weights, and average germination time	(Haghighi and Teixeira da Silva, 2014)
	*Salvia macrosiphon* Boiss*, Capsicum annuum L.* and *Festuca arundinacea* Schreb.	Germination rate, seedling length, and seedling fresh and dry weights	(Pourkhaloee et al., 2011)
	*Dodonaeaviscosa* L.	Germination rate, mean germination time, root and shoot lengths, and plant fresh and dry weights	(Yousefi et al., 2017)
	Tomato	Germination rate, seedling growth	(Khodakovskaya et al., 2010)

### Plant growth, photosynthesis, water uptake, and mineralnutrition applications

Nanomaterials can alter plant growth through nutrient uptake, improving capacity, resistance against biotic and abiotic stresses, promoting nitrogen metabolism, osmotic protection, and plant defense against pathogens. The conventional application of nutrients to the soil has several disadvantages in terms of nutrient availability to plants. Therefore, the foliar application is the most efficient method of compensating for nutrient deficiencies and increasing crop yield and quality. Foliar spraying also reduces environmental contamination and increases nutrient use efficacy by decreasing the amount of fertilizer applied to the soil. Biologically engineered ZnO ENMs have been used as a low-cost biomass-enhancing agent for crop plants (Venkatachalam et al., 2017). The effect of 25-200 mg/L of ZnO ENMs and phosphorous on cotton seeds was analyzed in growth stimulation experiments. Root and shoot lengths, total biomass, and the growth tolerance index were directly proportional to the concentration of (200 mg L^–1^) zinc oxide ENMs. Rizwan et al. (2019) addressed the effect of zinc and iron ENMs on wheat’s growth characteristics and capacity to accumulate cadmium (Cd) (*Triticum aestivum*). Wheat seeds were primed for one day with different concentrations of ZnO (25-100 mg L^–1^) and FeO (5-20 mg L^–1^). After priming, the seeds were grown in Cd-contaminated soil. Primed seeds produced more healthier plants than unprimed seeds as plant height, shoot and spike lengths, and plant dry weight were directly proportional to the concentrations of Zn and Fe ENMs. Sun et al. (2020) observed growth parameters of tomato plants sprayed with 20-100 mg L^–1^ ZnO ENMs; the foliar sprayed plants had greater plant heights, root lengths, and chlorophyll contents than the unsprayed plants. In addition, plant mineral and nutrient contents (calcium, magnesium, sucrose, starch and glucose) increased, significantly increasing iron deficiency tolerance.

**Figure 7:**
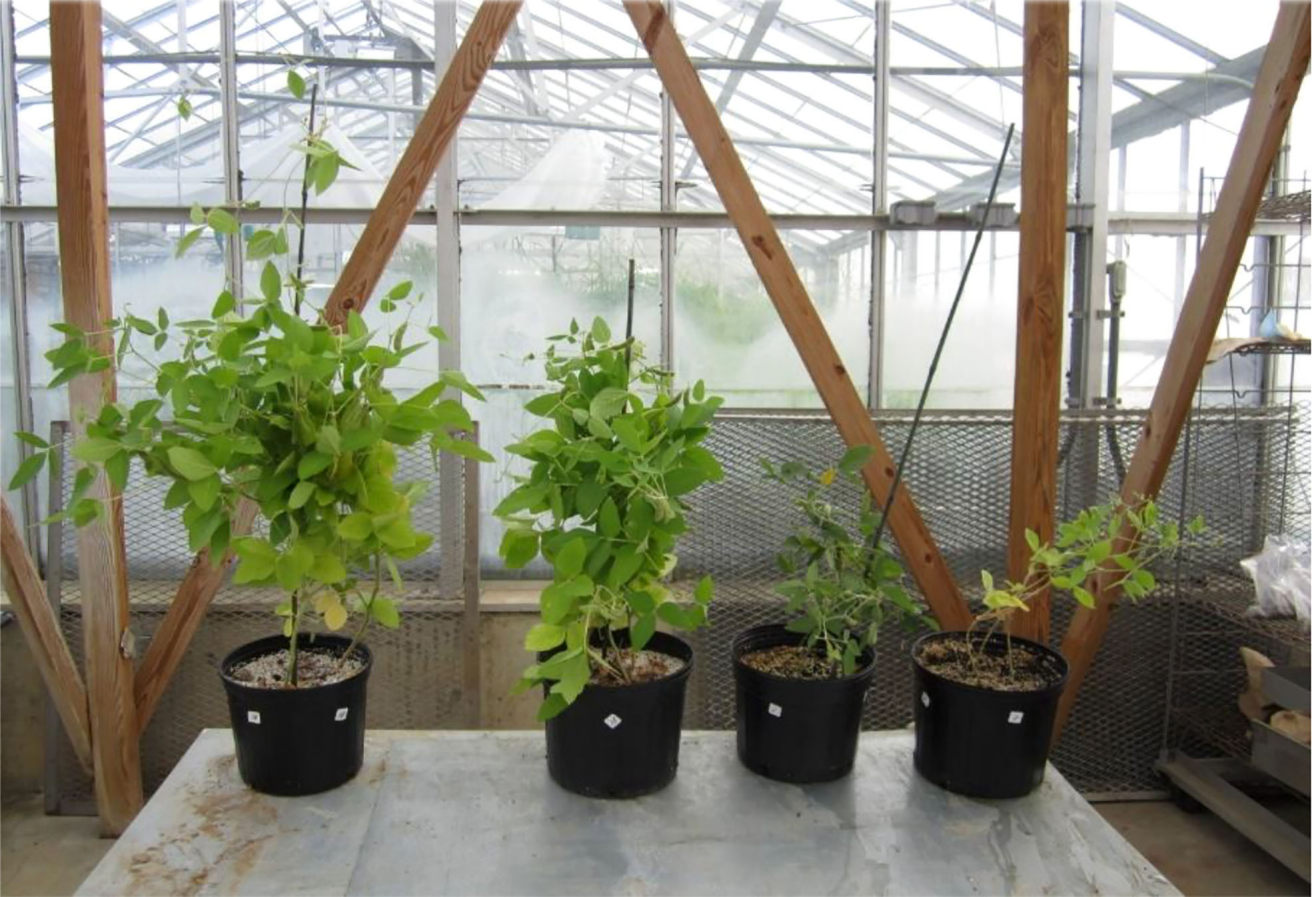
Soybean plant growth 15 weeks after germination under different fertilizer treatments (left to right) Hydroxyapatite ENMs (Engineered Nanomaterials), fertilizer plus normal phosphorous, fertilizer without phosphorous, and water (Liu and Lal, 2014). Copyright 2014.

Song et al. (2012) analyzed the effect of 10-2000 mg/L of Titanium dioxide (TiO_2_) ENMs and bulk TiO_2_ on the growth parameters of *Lemna minor*. For nanoparticle TiO_2_ concentrations <500 mg L^–1^, plant growth (root length, fresh weight, and frond number) had a directly proportional relationship with TiO_2_ concentration; a similar trend occurred for bulk TiO_2_ but to a lesser degree than the ENMs. The same growth parameters decreased at nanoparticle concentrations ≥500 mg L^–1^. Daghan (2018) analyzed the effect of TiO_2_ ENMs (25-100 mg L^–1^) on the seedling growth of maize irrigated with wastewater. The 25 mg L^–1^ treatment inhibited the adverse effects of wastewater and reached 100% seed germination, while higher doses had adverse effects on the seed germination rate. Liu and Lal (2014) used hydroxyapatite ENMs on soybean (see **Figure 7**). All growth parameters (growth rate, plant height, seed yield, aboveground and belowground biomass) increased compared to conventional phosphorus fertilizer.

Khan et al. (2021) analyzed the efficiency of silver ENMs (10-30 mM) for enhancing the growth of pearl millet under saline conditions (up to 150 mM NaCl). Adding ENMs under saline conditions significantly increased plant growth, including plant height, root, shoot, and dry grain weights, chlorophyll content, and photosynthetic rate. Suciaty et al. (2018) analyzed the effect of nano-silica (1.25-3.75 mL L^–1^) and rice hull ash (1-3 t ha^–1^) on the growth characteristics of *Grobogan*, a soybean cultivar. Rice hull ash increased plant height, but silica ENMs had no effect; silica ENMs and rice hull ash increased dry seed weight after applied individually. **Table 4** lists other studies using ENMs for plant growth applications.

**Table 4:** Application of engineered nano materials to enhance plant growth and other eco- physiological parameters. .

Nanoparticle (NP)	Plant species	Parameters analyzed	References
Zn-NP	*Oryza sativa*	Plant growth parameters, photosynthesis, antioxidant activity under cadmium stress	(Faizan et al., 2021)
	*Oryza sativa*	Translocation and deposition of ENMs and their effect on growth	(Afzal et al., 2021)
	Safflower	Change in antioxidant enzyme concentrations	(Hafizi and Nasr, 2018)
	Fenugreek (*Trigonella foenum-graecum*)	Plant growth parameters, including shoot length and dry weight, leaf quantity, nutrient percentage, chlorophyll content, and yield	(Sadak, 2019)
	*Zea mays*	Root and shoot growth	(Kumar et al., 2020)
	*Bacopa monnieri* (Linn.) Wettst.	Percentage of nutrients and antioxidant enzymes	(Krishnaraj et al., 2012)
	*Panax vietnamensis*	Number of somatic embryos	(Manh Cuong et al., 2021)
Ti-NP	Coriander	Plant height, fruit yield, and branch number	(Khater, 2015)
	Canola (*Brassica napus*)	Seed germination and seedling vigor	(Mahmoodzadeh et al., 2015)
	*Sorghum bicolor* (L.)	Seedling germination rate, gas exchange rate and root length	(Shoemaker and Wait, 2020)
Si-NP	Marigold (*Tagetes erecta* L.)	Plant height, branch number, and root length	(Attia and Elhawat, 2021)
	*Moringa oleifera L*	Plant growth parameters, including height, stem diameter, plant dry weight, leaf quantity and surface area	(Abou-Shlell et al., 2020)
	Basil (*Ocimum basilicum*)	Chlorophyll and plant biomass	(Lu et al., 2020)
	Maize (*Zea mays L*.)	Enzyme characteristics of soil, soil physical attributes, nutrient intake, and crop yield	(Osman et al., 2021)

### Plant disease control and fruit yield applications

**Figure 8:**
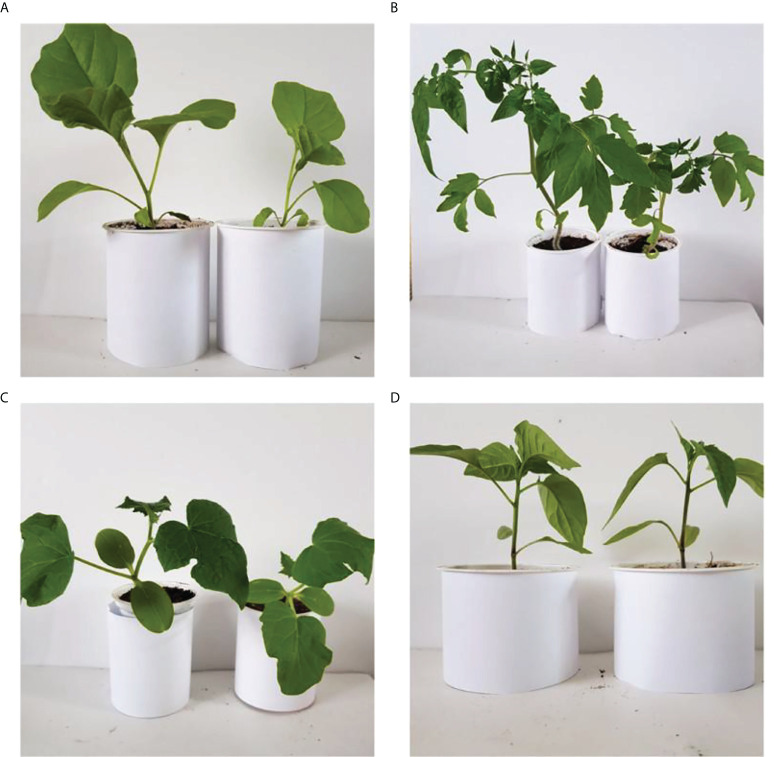
Effect of Se nano-fertilizers on **(A)** eggplant **(B)** cucumber **(C)** tomato, and **(D)** chili pepper (Gudkov et al., 2020). Copyright 2020.

Nanomaterials extensively used to alleviate plant diseases, as many nanomaterials have inhibitory effects against disease-causing microorganisms. Nanomaterials can also increase the yield of fruit-bearing plants by fulfilling their nutrient requirements or catalyzing photosynthesis. Hernández-Hernández et al. (2017) evaluated the effect of copper ENMs (0.02-10 mg) embedded in chitosan-polyvinyl alcohol (Cs-PVA) hydrogels on the growth, productivity, and fruit quality of tomato plants. Growth parameters (stem diameter, stem, root, and leaf weights, flower bunches per plant, leaf number per plant, and yield) increased with nanoparticle treatment. (Xing et al. 2021) observed dose-dependent antimicrobial activity of oleoyl-chitosan (O-chitosan) ENMs against several plant pathogenic fungi. Four fungal species (*N. sphaerica, B. dothidea, N. oryzae*, and *A. tenuissima*) had significant growth inhibition at 2 mg mL^–1^ O-chitosan. Sathiyabama and Manikandan (2018) Analyzed the efficacy of copper-chitosan ENMs (seed priming or foliar spray) on finger millet. The combined treatment i.e. seed priming and foliar spray, increased plant yield, chlorophyll content, and leaf quantity and decreased spore growth of *Pyricularia grisea*, which causes blast disease in millet. Hao et al. (2019) analyzed the efficacy of four engineered nanomaterials [multi-walled carbon nanotubes (MWCNTs), reduced graphene oxide (RGO), titanium dioxide (TiO_2_), and copper oxide (CuO)] for fungal disease control in rose plants. The ENMs were suspended in water (50 or 200 mg L^–1^) and sprayed on the leaves of rose plants infected with *Podosphaera pannosa* (*P. pannosa*), which causes powdery mildew disease (see **Figure 8**). After 19 days of 200 mg L^–1^ treatment, RGO, MWCNTs, and TiO_2_ ENMs had decreased the size of the infected area by 46%, 60%, and 70%, respectively. At 50 mg L^–1^, no inhibitory affect was observed for these three materials. For CuO ENMs, the 50 and 200 mg L^–1^ treatments decreased the infected area by 73% and 99%, respectively. Hao et al. (2018) analyzed the efficacy of four ENMs (TiO_2_, Ferric oxide (Fe_2_O_3_), MWCNT, and fullerene) for antiviral activity and growth of tobacco (*Nicotiana benthamiana*) infected with *turnip mosaic virus*. A 50 or 200 mg L^–1^ of each nanoparticle-water suspension was sprayed on tobacco leaves for 21 days. For the metallic ENMs, the maximum antiviral effect occurred at 50 mg L^–1^, while MWCNT had an inhibitory effect at both doses, while Fullerene (C_6_) had no antiviral activity. Similarly, the metallic ENMs increased fresh biomass by 56% at 50 mg L^–1^, with no change at 200 mg L^–1^. The 50 and 200 mg L^–1^ treatment of MWCNTs and C_60_ increased fresh biomass by 55%. Zahedi et al. (2019b) sprayed selenium ENMs embedded in sodium metal (Na_2_SeO_4_; 1-2 μM) on pomegranate leaves (*Punica granatum* L. cv. Malase Saveh) to evaluate their effect on growth characteristics and fruit quality. Plants sprayed with selenium ENMs had higher leaf surface area, chlorophyll content, and fruit numbers than unsprayed plants; the 2 μM treatment increased fruit yield/plant by 35%, peel thickness by 33.6%, fruit weight by 5.2%, fruit length by 30%, and fruit diameter by 11%, and decreased the fruit cracking percentage by 54%. Nandini et al. (2017) analyzed the efficacy of a foliar spray of selenium (mediated with *Trichoderma*) ENMs against downy mildew disease (*Sclerospora graminicola*) in pearl millet. Different species of *Trichoderma* were used; *T. asperelleum* combined with Se ENMs had the best results against downy mildew disease, with a MIC of 150 ppm. Similarly, seed priming with *T. asperelleum*-mediated Se ENMs improved plant height, tiller number per seedling, dry weight, and chlorophyll content. Joshi et al. (2021) used myogenic selenium ENMs to prime tomato seeds and analyzed their efficacy against late blight disease; the Se-primed seeds inhibited the disease by 73%, increased plant height by 51% and fruit weight by 83%, and expedited flower emergence from 70 days (unprimed) to 61 days.

Kumari et al. (2017) applied green-synthesized silver ENMs (5 μg mL^–1^) to tomato as an antidote for early blight disease (*Alternaria solani*). Three days of treatment eliminated the spore count. The silver ENMs treatment increased fresh and dry weights of leaves by 32 and 23%, respectively, Chlorophyll a by 14%, Chlorophyll b by 50%, total chlorophyll by 23%, and carotenoid content by 20%. Quiterio-Gutiérrez et al. (2019) sprayed selenium and copper ENMs on tomato leaves infected with *Alternaria solani*, which increased the number of fruit clusters per plant by 9%. **Table 5** lists other ENM applications for plant disease inhibition and fruit yield enhancement.

**Table 5:** Application of engineered nano-materials to enhance fruit yield and control disease in plants.

Nanoparticle (NP)	Plant species	Parameters	Reference
Cu-NP	Watermelon	Root and shoot length, fruit quantity, and leaf area	(González Gómez et al., 2017)
	Tomato (*Solanum lycopersicum* L.)	Fruit quantity and quality and antioxidant activity	(Hernández-Hernández et al., 2019)
Ti-NP	Tomato	*Xanthomonas perforans*, bacterial spot disease	(Paret et al., 2013)
	Tomato	Plant height, root length, seed germination, flower quantity, and fruit yield	(Raliya et al., 2015)
	*Beta vulgaris* L.	Effect against *Meloidogyne incognita*, *Pectobacterium betavasculorum*, and *Rhizoctonia solani*	(Afzal et al., 2021)
Se-NP	Eggplant, cucumber, tomato, chili pepper	Effect as fertilizer (see **Figure 8**)	(Gudkov et al., 2020)
	Strawberry (*Fragaria × ananassa* Duch.)	Salinity tolerance, H_2_O_2_ content	(Zahedi et al., 2019a)
	Pomegranate	Level of photosynthetic pigments, nutrient status, physical parameters (fruit cracking), phenolic content, osmolytes, antioxidant enzymes, abscisic acid, lipid peroxidation, and H_2_O_2_ content	(Zahedi et al., 2021)
	Bitter melon (*Momordica charantia*)	Nutrient content, leaf composition; effect on salt and temperature stresses	(Sheikhalipour et al., 2021)
	Faba bean	Antifungal activity against *Rhizoctonia solani* (root rot disease),	(Hashem et al., 2021)
C-NP	Bitter melon (*Momordica charantia*)	Number of produced flowers, time of fructifying	(Seddighinia et al., 2020)

## Limitations of using engineered nano materials

Despite many positive effects of using ENMs to enhance crop production, long-term exposure and accumulation of nanomaterials need to be addressed (Rajput et al., 2020). The effect of nanomaterials on plants largely depends on exposure intensity, concentration, size and shape of ENMs, and mode of contact (Corredor et al., 2009). Studies have shown that using ENMs in agriculture can affect plant metabolism, nutrient transport, and root morphology various cellular functions (Sunghyun et al., 2013). ENMs alter the protein, carbohydrate, and antioxidant patterns in some plants. Once ENMs enter cells. Adverse effects of ENMs include binding ENM ions to sulfhydryl groups of proteins and replacing essential cations with specific binding sites, leading to enzyme inactivation and production of reactive oxygen species, with consequent oxidative damage to lipids, proteins, and nucleic acids. ENMs can also trigger oxidative stress by depleting enzymes and major thiols containing antioxidant cells, disrupting the electron transport chain or inducing lipid peroxidation. Zhao et al. (2014) reported that CeO_2_ ENMs (800 mg kg^–1^) significantly reduced the phenolic content in cucumber, an important characteristic as it may reduce the risk of cancer. Parameters like the type of materials, synthesis method, Particle size and shape, functional groups stabilization agent used and application technique also affect the interactions and the outcome and add more complexity in cellular outputs (Bhardwaj et al., 2022). Detailed toxicological studies are required to analyze their safety for field use. An important concern regarding the use of nano-fertilizers is the buildup of ENMs in plant tissues without transformation and assimilation or even physical entrapment on plant surfaces. Stampoulis et al. (2009) investigated the effect of MWCNT, Ag, Cu, ZnO, and Si ENMs and their bulk materials (0, 1.0, 10, 50, 100, 500, and 1000 mg L^–1^) on the growth parameters of *Cucurbita pepo* (zucchini). The application of Cu ENMs at 1000 mgL^-1^ decreased root elongation by 77% compared to the control. At 1000 mg L^–1^, MWCNT, Ag, Cu, and ZnO ENMs decreased plant biomass by 60, 75, 90, and 90%, respectively, compared to control. Similar studies have reported potential adverse impacts of ENMs on plant growth, crop yield, shoot and root lengths, seed germination, water content, and relative chlorophyll (**Table 6**).

**Table 6:** Limitation and pitfalls of using engineered nanomaterials on plants.

Engineered nanomaterials	Concentration	Plant species	Adverse effects	References
Multiwalled carbon nanotubes (MWCNT) and oxidized-MWCNT	46×10^–3^ mg mL^–1^	Mustard (*Brassica juncea*) seeds	Reduced germination rate and plant growth	(Mondal et al., 2011)
Carboxy fullerenes (C_70_(C(COOH)_2_)_2_−4)	0−0.144 mg mL^–1^	Tobacco *(Nicotiana tabacum*) cells	Reduced plant growth	(Liu et al., 2013)
Water-soluble graphene	500–2000 mg L^–1^	cabbage (*Brassica oleracea)*, Tomato (*Solanum lycopersicum*), and spinach (*Amaranthus dubius*) plants	Reduced growth (Plant height and biomass)	(Begum et al., 2011)
Single-bilayer graphene oxide	0, 100, 200, 400, 800, 1600 mg L^−1^	Beans (*Vicia faba*)	Reduced plant growth and enzymatic activity	(Anjum et al., 2014)
Coated (humic acid) and uncoated zinc oxide (ZnO) ENMs	0, 10, 100, 1000 mg kg^–1^ soil	Cucumber plants	Reduced plant growth (root and shoot biomass)	(Moghaddasi et al., 2017)
Core-shell iron/iron oxide (Fe/Fe_3_O_4_) and copper-/copper oxide (Cu/CuO) ENMs	10, 20 mg L^–1^	Lettuce (*Lactuca sativa*)	Decreased plant growth, water potential, dry weight, and the concentration of several nutrients	(Trujillo-Reyes et al., 2014)
Cu/CuO and Zn/ZnO ENMs	0, 10, 50, 100, 500, 1000 mg L^–1^	Cucumber *(Cucumis sativus*)	Decrease seedling biomass at higher nanoparticle concentrations	(Kim et al., 2012)
Cu/CuO ENMs	0, 20, 80 mg kg^–1^	Cilantro (*Coriandrum sativum*)	Reduced germination, water content, and relative chlorophyll content	(Zuverza-Mena et al., 2005)
Cerium oxide (CeO_2_) and ZnO ENMs	400–800 mg kg^–1^	Cucumber (*Cucumis sativus*)	Decreased non-reducing sugars, affecting albumin and prolamin fractions	(Zhao et al., 2014a)
CuO ENMs	0, 50, 100, 200, 400, 500 mg L^–1^	Green pea (*Pisum sativum* L.)	Reduced plant growth (shoot and root lengths)	(Raliya et al., 2015)
Titanium dioxide (TiO_2_) (rutile) ENMs	0, 0.25, 0.5, 1.0, 1.5, 2.0, 2.5, 4.0, 6.0%	Spinach (*Spinacia oleracea*)	Reduced plant growth rate and crop yield	(Zheng et al., 2005)
CuO, aluminum oxide (Al_2_O_3_), and TiO_2_ ENMs	0, 20, 200, 2000 μg mL^−1^	Onion (*Allium cepa*)	Reduced root length	(Ahmed et al., 2018)
Fe₃O₄ and TiO₂ ENMs	(0, 100, 200 mg kg^–1^ soil)	Soybean (*Glycine max*)	Decreased plant growth	(Burke et al., 2015)
Titanium dioxide (TiO₂) ENMs	0, 10, 50, 100, 200, 1000, 2000 mg L^–1^	*Lemna minor*	Growth inhibited above 200 mg L^–1^	(Song et al., 2012)
Silver (Ag) and TiO_2_ ENMs	10, 20, 40 ppm Ag-NP and 31, 50, 100 ppm TiO_2_-NP	*Lemna (paucicostata)*	Decreased growth rate	(Kim et al., 2011)

## Conclusion and future prospectus

This review summarized eco-friendly and sustainable methods for synthesizing novel materials in agricultural applications and to exploit the chemical and physical properties of naturally occurring plant extract. Nanomaterials can be useful for specific applications using various natural extracts—easing the synthesis process by stabilizing and reducing agents—and in specific sizes and shapes. There are increasing concerns in the agricultural sector about enhancing crop production and yield while resisting harmful environmental stresses. Engineered nanomaterials (ENMs) for specific applications in the agricultural sector can be used as nano-fertilizers to provide essential nutrients for plant growth. They can also be used in seed priming and foliar spraying to mitigate environmental stresses and plant diseases. The increasing demand for food, medicine, and other crops and the changing climate requires new technologies to reduce the demand and supply gap. Several challenges are associated with the synthesis and application of ENMs, including determining the complete life cycle of ENMs in the food chain to identifying the potential toxicity mechanisms such as the uptake and accumulation of ENMs developing methodologies for green synthesis of ENMs at an industrial scale, exploiting new multifunctional bio-mediated ENMs with improved plant growth and control over environmental stresses, and identifying the effect of ENMs on physiological parameters and nutritional quality of different edible and non-edible plant species. Addressing these challenges will help decrease the reluctance of the agricultural sector to adopt new technologies.

## Author contributions

All authors contributed to the study’s conception and design. Material preparation, search, and collection of relevant articles and reviews were performed by KB, SH and FZ, ZA, HA. ZA, ZS and KHMS thoroughly checked the first draft and critically improved the manuscript. All authors contributed to the article and approved the submitted version.

## Conflict of interest

The authors declare that the research was conducted in the absence of any commercial or financial relationships that could be construed as a potential conflict of interest.

## Publisher’s note

All claims expressed in this article are solely those of the authors and do not necessarily represent those of their affiliated organizations, or those of the publisher, the editors and the reviewers. Any product that may be evaluated in this article, or claim that may be made by its manufacturer, is not guaranteed or endorsed by the publisher.

## Abbreviations

Ag, Silver; AgCl, Silver chloride; AgNO_3_, Silver nitrate; Cd, Cadmium; CdCl_2_, Cadmium chloride; CdTe, Cadmium telluride; CENMs, Carbon nanoparticles; Cu, Copper; CuO, Copper (II) oxide; CuSO_4_, Copper sulfate; DMF, Dimethylformamide; EMD, Electrolytic manganese dioxide; ENMs, Engineered nanomaterials; EPS, Exopolysaccharide; FeSO_4_, Ferrous sulfate; FeO, Iron oxide; Fe_3_O_4_, Iron (II,III) oxide; HCl, Hydrochloric acid; KCl, Potassium chloride; K_2_HPO_4_, Potassium hydrogen phosphate; MgSO_4_, Magnesium sulfate; MIC, Minimum inhibition concentration; MBC, Minimum bactericidal concentration; MHB, Muller-Hinton Broth; MRS, De Man, Rogosa, and Sharpe agar; MSA, Mercaptosuccinic acid; MWCNTs, Multiwalled carbon nanotubes; NaBH_4_, Sodium borohydride; NaCl, Sodium chloride; NaOH, Sodium hydroxide; Na_2_(PdCl_4_), Sodium tetrachloropalladate; Na_2_TeO_3_, Sodium tellurite; NPs, Nanoparticles; ORR, Oxygen reduction reaction; RGO, Reduced graphene oxide (RGO); RWC, Relative water content; Se, Selenium; SEM, Scanning electron microscopy; TEM, Transmission electron microscopy; TiO_2_, Titanium dioxide; XRD, X-ray diffraction; ZnO, Zinc oxide; ZrOCl_2_, Zirconyl chloride.
